# Refined study of the interaction between HIV-1 p6 late domain and ALIX

**DOI:** 10.1186/1742-4690-5-39

**Published:** 2008-05-13

**Authors:** Carine Lazert, Nathalie Chazal, Laurence Briant, Denis Gerlier, Jean-Claude Cortay

**Affiliations:** 1 Université Lyon 1, Centre National de la Recherche Scientifique (CNRS), VirPatH FRE 3011, Faculté de Médecine RTH Laennec, Lyon, France; 2 Université Montpellier 1, Université Montpellier 2, CNRS, Centre d'études d'agents Pathogènes et Biotechnologies pour la Santé (CPBS), UMR 5236, F-34965 Montpellier, France

## Abstract

The interaction between the HIV-1 p6 late budding domain and ALIX, a class E vacuolar protein sorting factor, was explored by using the yeast two-hybrid approach. We refined the ALIX binding site of p6 as being the leucine triplet repeat sequence (**L**xx)_4 _(**L**YP**L**TS**L**RS**L**FG). Intriguingly, the deletion of the C-terminal proline-rich region of ALIX prevented detectable binding to p6. In contrast, a four-amino acid deletion in the central hinge region of p6 increased its association with ALIX as shown by its ability to bind to ALIX lacking the proline rich domain. Finally, by using a random screening approach, the minimal ALIX_391–510 _fragment was found to specifically interact with this p6 deletion mutant. A parallel analysis of ALIX binding to the late domain p9 from EIAV revealed that p6 and p9, which exhibit distinct ALIX binding motives, likely bind differently to ALIX. Altogether, our data support a model where the C-terminal proline-rich domain of ALIX allows the access of its binding site to p6 by alleviating a conformational constraint resulting from the presence of the central p6 hinge.

## Background

A variety of enveloped viruses use for budding the host machinery that is required for the inward vesiculation of the membrane of the multivesicular bodies (MVB) [1]. For HIV-1 virus, this process is in part mediated through physical interactions between the viral Gag-p6 late domain and the host cellular factors Tsg101 (*t*umor *s*uppressor *g*ene 101) [2-6], and AIP1/ALIX (*ALG*-2 *i*nteracting protein X) [4,6].

In this context, the reduction of Tsg101 levels by siRNA or the introduction of a dominant-negative Tsg101 mutant severely blocks viral budding [7,8], while the disruption of the p6-ALIX interaction is less detrimental to HIV-1 budding. In EIAV, another member of the lentivirus subfamily of retrovirus, the Gag-p9 late domain contains a unique ALIX-binding motif (YPDL), which supports the release of virions in the absence of the Tsg101 cofactor.

Mechanistically, the interaction between p6 and Tsg101 is well-characterized: Tsg101 interacts with the p6 PTAP motif via its N-terminal UEV domain in a process that appears to be up-regulated when p6 becomes monoubiquitinylated at conserved Lys residues in positions 27 and 33 [3,7,9,10]. The structure of the Tsg101 UEV domain in complex with a 9-amino acid p6 peptide containing a central PTAP motif has been solved in solution by RMN [11,12].

The other p6-interacting partner, ALIX, which consists of 868 amino acids, is organized in three domains: (i) a N-terminal BroI domain responsible for CHMP4 recruitment in the endosomal pathway [13,14], (ii) a middle region (aa. 362–702), which interacts *in vivo *with p6 and p9 late domains [15], and (iii) a long C-terminal proline-rich region (PRR) that binds to Tsg101 [6]. Based on recent crystallographic data, the ALIX central region has been shown to adopt a "V" shape, which is the result of the complex arrangement of 11 α-helices with connecting loops that cross three times between the two arms of the V [16,17]. When overexpressed in mammalian cells, the V domain strongly inhibits HIV-1 particles release, and this inhibition is reversed by mutations of amino acid residues that specifically block binding of the ALIX V domain to p6 [18].

By using an *in vitro *pull-down approach, Strack *et al*., (2003) [4] have noted that the affinity of EIAV p9 for ALIX was significantly higher than that of HIV-1 p6. This suggests that the presence of a more efficient ALIX-binding site in p9 may compensate for the absence of a Tsg101 binding site. Such differences could be due in part to some intrinsic properties of the p6 polypeptide: (i) the p9 EIAV prototype motif L/IYPxL of different Gag late domains recognized by ALIX is only partially conserved in Gag-p6, where an adjacent LxxLF motif seems important for binding and, (ii) p6 adopts a random conformation in water without any preference for secondary structure [19]. However, under more hydrophobic conditions, i.e. in the presence of 50% aqueous TFE, p6 exhibits a functional helix-flexible-helix conformation, as assessed by its ability to bind to the Vpr protein [20].

The following work revealed that specific p6-ALIX association could be achieved through contacts between a minimal ALIX fragment containing amino acids residues 391–510 in the long arm of the V domain and a p6 late domain which has been mutated in its central hinge region. This mutant which displayed intermediary affinity for ALIX compared to HIV-1 p6 wild type and EIAV p9, suggests that in physiological conditions the constrained conformation of the HIV-1 late domain weakens its association with ALIX.

## Findings

### Yeast two-hybrid analysis of the HIV-1 p6-ALIX interaction

Several studies concerning the *in vivo *interaction between EIAV p9 and ALIX were previously designed using the Y2H assay [15,21]. For comparison, we examined for the first time the HIV-1 p6-ALIX interaction using a similar approach. Close characterization of the ALIX-binding site in HIV-1 p6 was accomplished by systematically introducing alanine mutations at every amino acid residue contained within the p6 minimal region (aa: 31–46) that had been previously implicated in ALIX recognition [4]. These Gal4 DBD-p6 bait constructs were individually co-transformed into the yeast strain AH 109 with a prey plasmid encoding the ALIX protein (868 amino acid-long) fused to the Gal4 AD. Relative quantification of the protein/protein interaction strength was monitored by measuring the β-galactosidase activity in yeast cells cotransformed with bait and prey expressing plasmids.

**Figure 1:**
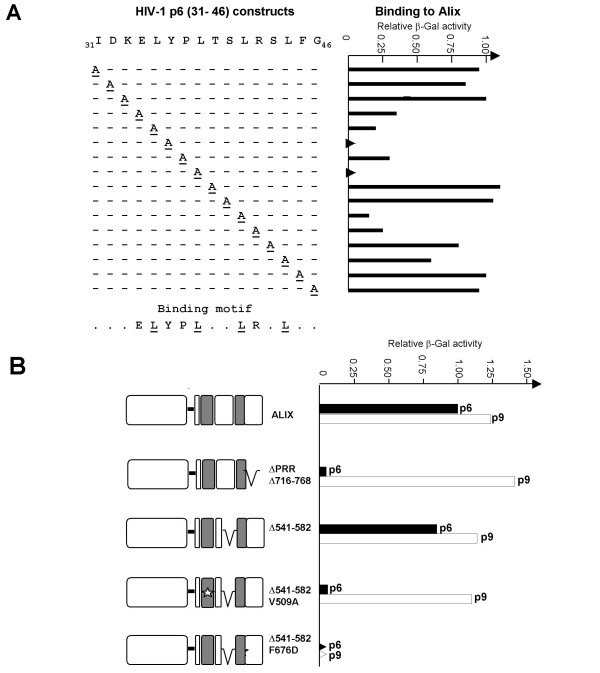
**Yeast two hybrid interaction between the HIV-1 p6 late domain and ALIX**. (A) Alanine-scanning mutagenesis of the ALIX-binding region of p6. The HIV-1 p6 (pNL4-3, NIH AIDS Research and Reference Reagent Program) derived-DNA fragment was generated by PCR and inserted in frame with the Gal4-DBD of pGBKT7 (Clonetech). ALIX was PCR-generated from plasmid pGAD AIP-1/ALIX [23] and fused in frame with the Gal4 AD of pACT2 (Clontech). Yeast strain AH109 (MATa, trp-901, leu2-3, 112, ura3-52, his3-200, gal4Δ, gal80Δ, LYS2::GAL1UAS-GAL1TATA-HIS3, GAL2UAS-GAL2TATA-ADE2; URA3::MEL1UAS-MEL1TATA-lacZ, MEL1) was cotransformed with pGBKT7 and pACT2 derivatives. The relative strength of the protein interaction between bait and prey was determined in yeast transformants grown at 30°C in SD/-Leu/-Trp selection medium by measuring β-galactosidase activity according to the protocol described in the Yeast β-Galactosidase Assay Kit from Pierce. Values are referred to 100% β-galactosidase activity measured in yeast cells cotransformed with wild-type p6 and ALIX proteins. Liquid culture assays were performed in triplicate. In the histogram, the lack of activity is indicated by triangles. (B). ALIX and fragments thereof were tested for interaction with Gal4 DBD-HIV-1 p6 and/or GAL4 DBD-p9 EIAV_UK _[33]. *Bro1: *Bro1-rhophilin-like domain; *PRR*: proline-rich region. Deletions and point mutations were generated using a splice-overlap extension method [34]. A reference value of 1 was set to β-galactosidase activity resulting from the interaction of ALIX with either p9 or p6 wild-type proteins. The lack of activity is indicated by triangles.

As shown in Figure 1A, the alanine scan clearly revealed that both amino acid residues in the YPx_n _L consensus sequence as well as the leucine triplet repeat sequence (Lxx)_4 _are crucial for HIV-1 p6 to interact with ALIX. This motif overlaps completely with the helix-2 in p6 as identified by NMR analysis [20], thus indicating that the ability of complex formation *in vivo *closely depends on the complete integrity of the secondary structure of helix-2. In details, the alanine substitution has variable effect from complete abolition of binding for Y36A and L38A, severe reduction in binding for E34A, L35A, P37A, L41A, R42A and a moderate but significant reduction for L44A, while the substitution of all other residues were well tolerated. Collectively, the binding data of our p6 mutants, are in full agreement with experimental data obtained *in vitro *with p6-derived peptides [18], except for the poor binding activity of L35A mutant, that has not been previously found in an *in vitro *binding assay measured by SPR [16-18]. However, the same authors reported that the corresponding L22A mutation in p9, completely abrogates p9 binding to ALIX. Thus, both L35 in p6 and L22 in p9 late domains are critical residues in the binding to ALIX.

In subsequent experiments, we tested in the Y2H system the potential interaction between the p6 domain and different ALIX mutant constructs. Side-by-side comparison was carried out in the presence of the EIAV p9 domain. Unexpectedly, a truncation of the proline-rich region in ALIX (ALIX_ΔPRR_) from amino acids 716 to 868 impaired ALIX binding to p6 *in vivo*, while p9 still bound to the ALIX mutant (Fig. 1B). Because *in vitro *ALIX deleted from PRR has a lower affinity for p6 than for p9 (dissociation constants measured by SPR are 60 μM and 1.2 μM, respectively) [16], our data point out to a major role of PRR as positive regulator for the ALIX-p6 interaction.

The region 541 to 582 contains essentially helix-7 residues in the arm 1 of the V domain (nomenclature is from [17]) and could play a key role in ALIX oligomerization. Indeed, bioinformatic analyses using the MultiCoil prediction program [22] suggested that this region in ALIX has a high probability for forming a trimeric coiled-coil (with a maximum trimeric residue probability value of 0.691 for S575). Y2H analysis of ALIX_Δ541–582 _(Fig. 1B) provides evidence that helix 7 (and probably oligomerization of ALIX protein) is dispensable for interaction with p6 and/or p9 *in vivo*.

From structural studies, the ALIX viral late domain-binding site has been mapped to a large hydrophobic pocket on the long arm of the V domain. Mutational experiments targeting amino acid residues, which form the surrounding walls, revealed in particular that substitutions V509A in α5 and F676D in α11 caused a dramatic effect on the ability of the protein to bind a p6-derived peptide *in vitro *[17].

The effect of these two mutations on interaction with p6 and p9 was evaluated *in vivo *using the Y2H assay. As expected, both the V509A and F676D mutations prevented the yeast cell growth on selective medium when tested against the bait-p6 protein (Fig. 1B). Quite different results were obtained with p9, since the V509A mutation was well tolerated. Taken together, these results are consistent with a model in which the intact conformation of the binding site is required for the efficient interaction between ALIX and the helix-2 amino acid residues in the HIV-1 p6 late domain. In this regard, it has been postulated that p6 may bind coaxially to the V domain hydrophobic pocket and form a four-helix bundle together with ALIX α- 4, α- 5 and α- 11 [17]. The molecular mechanisms by which p9 binds to ALIX are likely involving a less stringent process in terms of structural requirement and integrity of the late domain binding site. Indeed, the short YPDL tetrapeptide motif detected in p9 constitutes a specific binding epitope for AIP1 family members throughout the eukaryotic evolution [23]. Moreover, this motif appears very stringent since the close YLDL motif within the Sendai virus M protein, binds to the Bro1 domain of ALIX between amino acid residues 1–211, i.e. outside of the p9 binding domain [24].

### Description of a HIV-1 p6 mutant with increased ALIX-binding affinity

**Figure 2:**
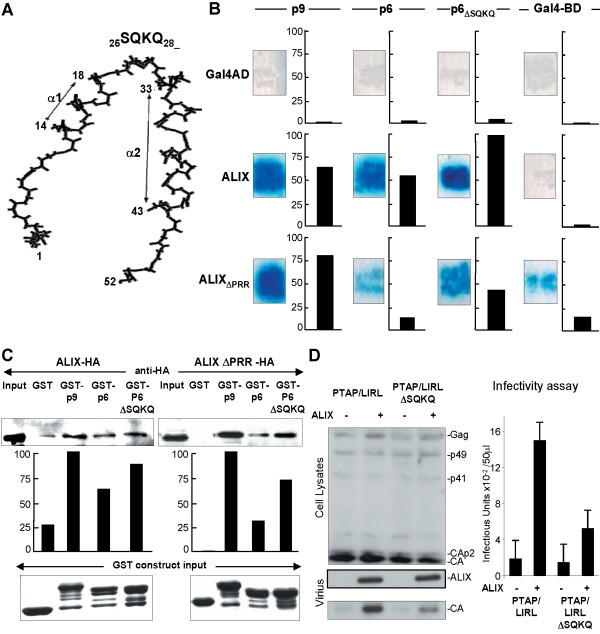
**Characterization of the HIV-1 p6_ΔSQKQ_-ALIX interaction**. (A) HIV-1 p6 (1–52) structure according to [20]. (B) HIV-1 p6 (1–52), p6 _ΔSQKQ _and EIAV p9 interaction with either full length ALIX, or ALIX_ΔPRR _as determined in yeast two-hybrid assay and revealed by α-galactosidase expression quantified by densitometry. Data are expressed as percentage of the maximal activity observed after the cotransformation with mutant p6_ΔSQK _and ALIX proteins. (C) Interaction determined by GST-pull-down. GST fusion proteins were obtained by subcloning of HIV-1 p6, p6_ΔSQKQ _and EIAV p9 domains into pGEX-KT (GE Healthcare). Purified GST-proteins bound to glutathione-beads were mixed with cell lysates containing either ALIX-HA or ALIX_ΔPRR_-HA proteins. Co-precipitated proteins were detected by western blotting using an anti-HA monoclonal antibody (Clone HA.11) and quantified by densitometry. Results were expressed in percentage of the band intensity measured in the presence of the GST-p9 construct. Equivalent loads of the GST fusion proteins were verified by Coomassie blue staining of the glutathione-bound fraction. The lanes marked *Input *contain 10% of the cell extract used for binding experiments. (D) L-domain function as determined using a complementation assay [6, 20, 21, 35]. 293T cells were cotransfected with 300 ng of HIV proviral plasmid (Nlδp6) that lacks the p6 L domain, 200 ng of plasmid expressing a truncated HIV Gag protein (Gagδp6) fused to the p6 domain of Gag mutated on the PTAP L domain (PTAP/LIRL) or to the p6 PTAP/LIRL_ΔSQKQ _and 200 ng of plasmid expressing myc tagged ALIX (1–868) or an empty vector. Virion samples pelleted through 20% sucrose cushions, Gag expression and Myc-ALIX were analyzed [21] by western blotting with a mouse antibody anti-HIV CAp24 serum (Biodesign International) and with a monoclonal antibody anti-Myc (Santa Cruz Biotechnology). Virion was also measured 48 h later using an infection assay with MAGIC-5B (HeLa-CD4/CCR5 LTR-*lacZ*) indicator cells for HIV-1. Error bars in infectivity assays represented standard deviations of three separate experiments.

Isothermal titration calorimetry experiments performed on the HIV-1 p6-derived peptide (DKELYPLTSLRSLFGN) and the EIAV p9-derived peptide (QTQNLYPDLSEIKKE) have reported that both peptides interacted *in vitro *with ALIX with quite similar *K*_*d *_values [18], while full-length p6 displayed a much lower ALIX-binding affinity when compared to p9 [16]. A possible explanation for such divergent behaviour is that p6 could exhibit a constrained conformation for ALIX binding. Analysis of the high resolution structure of p6 [20] (Fig. 2A) suggests that the hinge region (aa: 19–32) in the vicinity of the ALIX-binding site (helix α- 2) may play such a structural function.

Therefore a p6 mutant deleted for amino-acids S25 to Q28 (see location in Fig. 2A) referred as p6_ΔSQKQ _was produced and was tested for interaction with ALIX by the Y2H assay. p9-ALIX, p6-ALIX, and p6_ΔSQKQ_-ALIX gave rise to detectable growth on selective media when incubated for 3 days at 30°C, indicating that protein-protein interaction has occurred (Fig. 2B). The binding affinity quantified by in situ α-galactosidase staining using X-α-Gal as a substrate revealed a quite stronger interaction between p6_ΔSQKQ_-ALIX as compared to p6-ALIX and/or p9-ALIX. When tested for binding to the truncated ALIX_ΔPRR_, the p6_ΔSQKQ _mutant supported significant growth on selective media. α-galactosidase staining was however reduced as compared to that observed in yeast co-expressing p9. Under similar conditions, the p6-ALIX_ΔPRR _cotransfectants were found unable to grow as expected from data described in Figure 1. The absence of a significant growth on selective media of yeast co-expressing p9-Gal4AD, p6-Gal4AD, p6_ΔSQKQ_-Gal4AD and ALIX-Gal4DBD ruled out the possibility that the different bait and prey proteins tested could directly activate the Gal4 responsive promoter and thus validated the specificity of the above described interactions.

To confirm these data, GST-pull down assays were then carried out with extracts from cells expressing either ALIX-HA or ALIX_ΔPRR_-HA. This truncation was used because the removal of the proline-rich region has been described to improve the efficiency of *in vitro *interaction [4]. After their expression in *E. coli*, the following fusion proteins, GST, GST-p6, GST-p9 and GST-p6_ΔSQKQ _were bound to glutathione-Sepharose beads, and allowed to interact with either ALIX-HA or ALIX_ΔPRR_-HA. After extensive washings, the complexes were eluted, subjected to electrophoresis under denaturing conditions, transferred to a PVDF membrane and reacted with an anti-HA monoclonal antibody. As shown in Figure 2C, the three GST constructs bound to both ALIX-HA and ALIX_ΔPRR_-HA proteins in the following strength order: GST-p9>GST-p6_ΔSQKQ_>GST-p6 while the control GST displayed no detectable binding activity.

The overexpression of wild type ALIX has been shown to partially rescue budding defects of HIV-1 particles with a p6 domain containing mutations in the PTAP motif (called PTAP/LIRL), i.e. unable to recruit the ESCRT I component Tgs101 [17,21]. We therefore tested the ability of ALIX to alleviate the release defect of HIV-1 PTAP/LIRL mutated viruses containing or not the SQKQ deletion. We used a previously described complementation assay [6,21]: HIV-1 proviral plasmid (NLδp6) that lacks the p6 domain was cotransfected into 293T with a plasmid expressing a truncated HIV-1 Gag protein (Gagδp6) fused to either the PTAP/LIRL p6 domain or the PTAP/LIRL p6_ΔSQKQ _domain of HIV-1, together with an expression vector for ALIX or empty vector. As shown in Figure 2D, the coexpression of ALIX led to an increase in viral particle production and infectivity by both HIV-1 PTAP/LIRL p6 virus and HIV-1 PTAP/LIRL p6_ΔSQKQ_. Similar effect of ALIX on PTAP/LIRL p6_ΔSQKQ _was observed, although with reduced efficiencies. In summary, the deletion amino acid residues located in the p6 hinge region (ΔSQKQ) enhanced binding to ALIX, and partially allowed the rescue of HIV-1 PTAP/LIRL upon ALIX overexpression. This limited enhancing effect of this deletion on HIV-1 PTAP/LIRL p6 upon ALIX overexpression is indicative of a negative modulation played by the hinge region of HIV-1 p6. This negative modulation would be part of the highly complex process that optimises the HIV-1 budding.

### Mapping of a minimal p6_ΔSQKQ _binding site within the middle region of ALIX

**Figure 3:**
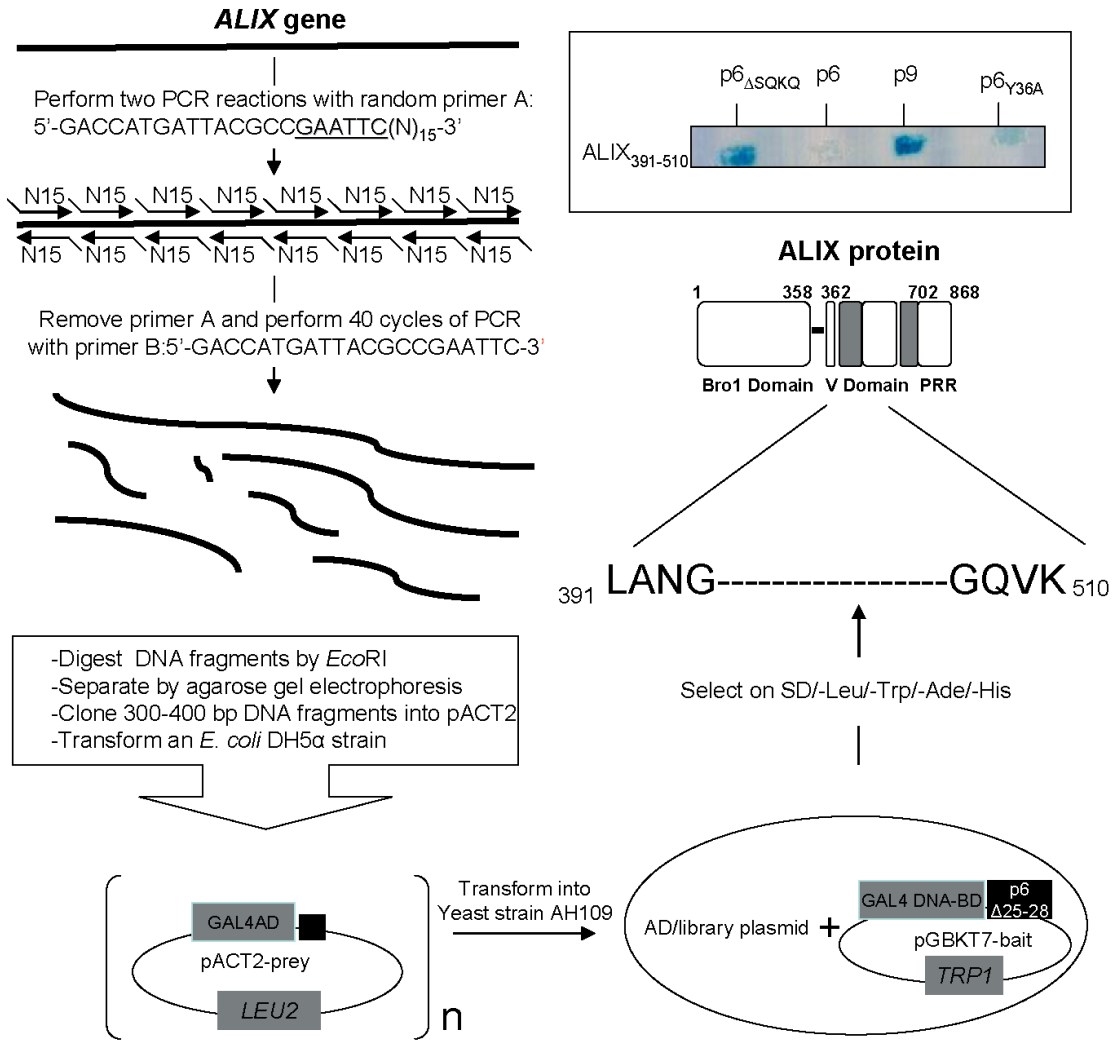
**Y2H-TPCR screening assay used to map the HIV-1 p6_ΔSQKQ_-binding site in ALIX**. Random tagged PCR was performed using full length AIP-1 DNA sequence as a template according to a previously described technique {Chen, 2005 #33}. The resulting library of AIP-1 fragments was amplified in *Escherichia coli *DH5α. The ALIX library was cotransformed with pGBKT7-p6_ΔSQKQ _bait into AH109 yeast and streaked onto SD/-Ade/-His/-Leu/-Trp plates. Clones growing on selective plates after 4–5 days at 30°C were recovered by transformation into bacteria, and inserts were sequenced. The amino acid sequence of ALIX (aa: 391–510, REFSEQ: accession NM_013374.3) that is represented, corresponds to the p6_ΔSQKQ_-binding fragment identified in this work.*Inset*: p6, p6_Δ*SQKQ*, _p6 Y36A and p9 were tested for interaction with ALIX_391–510 _in experimental conditions similar to those described in Figure 2B.

To isolate a minimal region in ALIX that was still able to bind to p6_ΔSQKQ _we used a previously described Y2H assay called Y2H-TPCR [25] (Fig. 3). Briefly, a library of random ~300 bp long PCR fragments derived from the ALIX cDNA was subcloned downstream to the Gal4 AD, and screened for potential interaction against the Gal4 DBD/p6_ΔSQKQ _bait. After selection on selective SD/-Trp/-Leu/-His/-Ade medium, one ALIX fragment encompassing residues 391–510 was found to bind to p6_ΔSQKQ_. This fragment also interacted with p9, but not with p6 or with the double mutant p6_ΔSQKQ _Y36A unable to bind to ALIX as reported above, thus demonstrating that the interaction was specific (Fig. 3*inset*). It is worth noticing that ALIX_391–510 _fragment partially rebuilds the arm 2 of the V-shape domain and encompasses the great majority of the hydrophobic surface residues which presumably contact the late domains [17]. Remarkably, the minimal p6 and p9 binding site ALIX_391–510 _that we identified are present in the truncated ALIX_409–715_[15], ALIX_364–716_, and ALIX_1–503 _[21] fragments known to bind the YPDL motif.

## Conclusion

If HIV-1 budding process is dependent on the presence of both Tsg101 and ALIX proteins, ALIX recruitment by the p6 late domain occurs at relatively low levels. On an evolutionary point of view, it has been proposed [26] that a strong ALIX-binding site in combination with a Tsg101-binding site may confer a disadvantage to HIV-1 perhaps because hyperactivation of ALIX can lead to apoptosis [27]. We identified here what makes p6 a weak ALIX-binding factor. The ALIX-binding site in p6 includes the consensus YPxnL sequence inserted into a leucine triplet repeat motif (Lxx)4. By contrast to p9, p6 is unable to bind to a detectable level to a truncated form of ALIX deleted from its PRR (aa: 716–868). Beside the essential role of the C-terminus of the ALIX PRR in recruiting the ESCRT machinery to promote HIV-1 budding [28], our data support that the PRR could also facilitate the recruitment of p6. The distinct behaviour of the p6_ΔSQKQ _mutant, which still binds to ALIX_ΔPRR_, sheds some light on a particular structural aspect of p6. Indeed, analyzing HIV-1 subtypes sequenced until now, the p6 domain appears by far the most variable domain in the Gag polyprotein precursor and natural deletions or insertions are frequently observed in the central region of p6 between S14 and I31 [29]. Interestingly, mutation of the 27KQE29 motif has never been observed so far. If K27 residue in this motif is a substrate for ubiquitin modification [30], it is unclear whether Gag itself needs to be ubiquitinylated for budding. The hinge region of p6 adopts a constrained conformation, which prevents optimum binding to ALIX. The deletion of the hinge region that encompasses the highly conserved KQE motif results in an increased affinity of the mutant late domain for ALIX probably by alleviating the bend between N and C terminus of p6. As suggested by *in vivo *analysis, a tightly interaction between late domain inhibited partially rescue of particle production upon ALIX over-expression. We can speculate that the ALIX-binding site is not necessarily optimized for high-affinity particularly in the context of HIV-1 which employs two late domains. Taken together, these observations point out that the negative activity of the p6 hinge may provide an additional ALIX-dependent regulatory process in the mechanisms that control HIV-1 budding, the complexity of which is far from being fully understood as shown by the recent finding of nucleocapsid binding to ALIX [31]. Finally, by using a random strategy, we have refined the p6_ΔSQKQ _and p9 binding site down to the ALIX 391–510 fragment. Furthermore, from our data, both HIV-1 p6 and EIAV p9 bind to an overlapping site on ALIX but in a quite different way. If the interaction between ALIX and p9 is direct, that of p6 to ALIX occurs in two steps. We propose that the PRR domain of ALIX could first contact p6 so as to alleviate the conformational constraints of the p6 hinge region and enable the subsequent binding of the HIV-1 late domain to the ALIX V domain within the 391–510 fragment.

During the submission of this work the crystal structures of ALIX V domain in complex with short peptides spanning the HIV-1 and EIAV late-domain motifs was reported [32]. Because p6 and p9 peptides, but not the full-length proteins, bind ALIX V domain with similar affinities, the authors proposed that interactions of ALIX with full-length p6 and p9 are regulated by subtle protein context-dependent effects. Our work based on Y2H experiments provides further support to biosensor experiments reported by Zhai et al. [32] and validates a model in which the structural constraints in the hinge region of p6 weaken the binding of the HIV-1 late domain to ALIX. Accordingly, the interaction of p6 late domain with ALIX appears to be a finely tuned process required for optimal budding of HIV-1.

## Abbreviations

Aa: amino acid; AD: activation domain; Bp: bp; DBD: DNA binding domain; EIAV: equine infectious anemia virus; HIV-1: human immunodeficiency virus type-1; PMSF: phenylmethanesulphonylfluoride; SD: synthetic dropout; SPR: surface plasmon resonance; X-α-Gal: 5-Bromo-4-Chloro-3-indolyl α-D-galactopyranoside; Y2H: yeast two-hybrid.

## Competing interests

The authors declare that they have no competing interests.

## Authors' contributions

DG, NC, LB and J-CC have conceived the study and analyzed data. J-CC, NC and CL performed the laboratory work and wrote the manuscript. CL and NC equally contributed to this work. All the authors have read and approved the manuscript
